# Supplementary material for: Yolk Fatty Acid Content, Lipid Health Indices, and Oxidative Stability in Eggs of Slow-Growing Sasso Chickens Fed on Flaxseed Supplemented with Plant Polyphenol Extracts
Source: Foods. 2023 Apr 27;12(9):1819. doi: 10.3390/foods12091819 (PMC10178081; doi:10.3390/foods12091819)
Supplement: Supplementary file 1 [file foods-12-01819-s001.zip › Table S1.pdf]

Table S1. The effects of feeding plant polyphenol extracts and flaxseed on fat content and fatty acid concentrations in the raw egg yolks of slow growing Sasso chickens

Fatty acids (mg/g)	[1]Treatments										Random effect	p-value
	FS		VE8		DA8		TS8		CD8			
	Mean	95% [LCL, UCL]	Mean	95% [LCL, UCL]	Mean	95% [LCL, UCL]	Mean	95% [LCL, UCL]	Mean	95% [LCL, UCL]		
C14:0	0.80	[0.72, 0.86]	0.78	[0.70, 0.85]	0.82	[0.74, 0.89]	0.77	[0.69, 0.84]	0.80	[0.73, 0.87]	non 0	0.8556
C15:0	0.15	[0.13, 0.16]	0.17	[0.15, 0.18]	0.16	[0.14, 0.17]	0.18	[0.16, 0.19]	0.16	[0.144, 0.17]	non 0	**0.2704**
C16:0	63.9	[61.1, 66.7]	65.2	[62.4, 68.0]	66.3	[63.6, 69.1]	64.0	[61.3, 66.8]	65.5	[62.7, 68.3]	non 0	0.6833
C17:0	0.49	[0.43, 0.53]	0.55	[0.50, 0.60]	0.50	[0.45, 0.55]	0.58	[0.53, 0.63]	0.51	[0.45, 0.55]	non 0	0.06333
C18:0	22.7	[21.6, 23.9]	21.5	[20.3 , 22.6]	22.2	[21.0, 23.3]	**23.1**	[21.9 , 24.2]	22.7	[21.5 , 23.9]	0	0.33209
C20:0	0.07	[0.06, 0.08]	0.07	[0.07, 0.08]	0.08	[0.07, 0.08]	0.08	[0.07, 0.08]	0.08	[0.07, 0.08]	non 0	0.5951
C23:0	0.07	[0.05, 0.09]	0.08	[0.06, 0.09]	0.09	[0.07, 0.11]	0.08	[0.06, 0.10]	0.08	[0.07, 0.11]	non 0	0.5100
C14:1cis-9	0.15	[0.12, 0.18]	0.14	[0.11, 0.17]	0.17	[0.14, 0.20]	0.14	[0.11, 0.17]	0.16	[0.13, 0.19]1	non 0	0.4706
C16:1cis-9	6.97	[6.17, 7.78]	6.68	[5.87, 7.49]	7.57	[6.76, 8.38]	6.58	[5.77, 7.39]	7.31	[6.50, 8.12]	non 0	0.3638
C18:1trans-9	0.19	[0.16, 0.22]	0.21	[0.18, 0.24]	0.22	[0.18, 0.24]	0.22	[0.18, 0.25]	0.22	[0.18, 0.25]	non 0	0.7892
C18:1cis-9	105	[101, 109]	104	[100, 108]	105	[101, 109]	105	[101, 109]	106	[102, 110]	0	0.9835
C18:1trans-11	0.22	[0.20, 0.24]	0.19	[0.17, 0.21]	0.18	[0.16, 0.20]	0.19	[0.17, 0.21]	0.19	[0.17, 0.21]	0	0.0789
C18:1cis-11	4.75	[4.43, 5.07]	4.62	[4.30, 4.93]	4.84	[4.53, 5.16]	4.72	[4.40, 5.04]	4.93	[4.61, 5.25]	non 0	0.6624
C18:2*n*-6	33.7	[30.9, 36.5]	37.2	[34.4, 40.1]	35.6	[32.8, 38.4]	38.8	[36.0, 41.6]	36.2	[33.4, 39.1]	non 0	0.1383
C18:3n-6	0.166	[0.15, 0.18]	0.19	[0.17, 0.20]	0.18	[0.16, 0.19]	0.19	[0.17, 0.20]	0.18	[0.16, 0.19]	non 0	0.1922
C18:3*n*-3	6.60	[5.39, 7.80]	6.56	[5.36, 7.77]	7.63	[6.43, 8.83]	8.51	[7.31, 9.72]	8.08	[6.88, 9.28]	non 0	0.0981
C18:2cis 9-trans-11	0.16	[0.12, 0.19]	0.15	[0.11, 0.18]	0.18	[0.15, 0.22]	0.19	[0.16, 0.22]	0.15	[0.11, 0.18]	non 0	0.1649
C18:4n-3	0.07	[0.05, 0.08]	0.07	[0.05, 0.08]	0.07	[0.06, 0.08]	0.09	[0.07, 0.10]	0.08	[0.07, 0.09]	non 0	0.2717
C20:2n-6	0.29	[0.25, 0.32]	0.32	[0.28, 0.35]	0.31	[0.27, 0.34]	0.33	[0.29, 0.36]	0.31	[0.28, 0.34]	non 0	0.6406
C20:3n-6	0.34	[0.31, 0.37]	0.34	[0.31, 0.36]	0.35	[0.32, 0.37]	0.35	[0.32, 0.37]	0.36	[0.33, 0.39]	non 0	0.7336
C20:3n-9	0.06	[0.04, 0.07]	0.06	[0.04, 0.07]	0.06	[0.04, 0.07]	0.07	[0.05, 0.08]	0.05	[0.04, 0.06]	non 0	0.7791
C20:3n-3	0.13	[0.10, 0.14]	0.12	[0.10, 0.14]	0.15	[0.13, 0.17]	0.15	[0.13, 0.17]	0.15	[0.13, 0.17]	non 0	0.05859
C20:4*n*-6	3.79	[3.52, 4.07]	4.09	[3.81, 4.37]	3.65	[3.37, 3.93]	4.09	[3.81, 4.37]	4.00	[3.73, 4.28]	non 0	0.1045
C20:5*n*-3	0.16^{a}	[0.14, 0.18]	0.16^{a}	[0.13, 0.17]	0.20^{b}	[0.17, 0.22]	0.21^{b}	[0.18, 0.23]	0.21^{b}	[0.19, 0.23]	0	0.00035
C22:4n-6	0.41	[0.34, 0.48]	0.48	[0.41, 0.55]	0.47	[0.39, 0.53]	0.43	[0.36, 0.50]	0.42	[0.35, 0.49]	non 0	0.5725
C22:5*n*-6	0.38	[0.19, 0.57]	0.45	[0.26, 0.64]	0.33	[0.14, 0.52]	0.24	[0.04, 0.42]	0.24	[0.05, 0.43]	non 0	0.4092
C22:5*n*-3	0.81^{a}	[0.60, 1.02]	0.95^{a}	[0.75, 1.16]	1.29^{b}	[1.08, 1.50]	1.07^{ab}	[0.86, 1.27]	1.08^{ab}	[0.87, 1.29]	non 0	0.0338
C22:6*n*-3	5.96^{a}	[5.51, 6.42]	6.31^{ab}	[5.86, 6.77]	6.50^{ab}	[6.04, 6.95]	7.39^{c}	[6.93, 7.84]	6.93^{cb}	[6.47, 7.38]	non 0	0.00118
[2]∑SFA	88.2	[84.9, 91.6]	88.4	[85.1, 91.7]	90.2	[86.9, 93.5]	88.8	[85.5, 92.1]	89.9	[86.5, 93.2]	non 0	0.8785
[3]∑MUFA	117	[113, 122]	116	[112, 120]	118	[114, 122]	117	[113, 121]	119	[115, 123]	0	0.9255
[4]∑ PUFA	53.1^{a}	[49.2, 56.9]	57.5^{ab}	[53.6, 61.3]	57.0^{ab}	[53.1, 60.8]	62.1^{b}	[58.3, 66.0]	58.5^{b}	[54.7, 62.4]	non 0	0.0366
∑ *n*-3 PUFA	13.7^{a}	[12.1, 15.3]	14.2^{a}	[12.6, 15.8]	15.8^{ab}	[14.2, 17.4]	17.4^{b}	[15.8, 19.0]	16.5^{b}	[14.9, 18.1]	non 0	0.01226
∑ *n*-6 PUFA	39.1	[36.3, 41.9]	43.1	[40.3, 45.9]	40.9	[38.0, 43.7]	44.5	[41.6, 47.3]	41.8	[38.9, 44.6]	non 0	0.0935
Fat (%)	25.9	[25.2, 26.5]	26.2	[25.5, 26.9]	26.5	[25.8, 27.2]	26.8	[26.1, 27.5]	26.7	[26.0, 27.4]	non 0	0.2900

Estimated marginal means (emmeans) with 95% upper confidence (UCL) and lower confidence limits (LCL); emmeans within a row with ^{abcd} different superscript letters are significantly different at $p < 0.05$. [1]FS: 75 g flaxseed + no antioxidant sources, VE8: 75 g flaxseed + 800 mg α-tocopherol/kg, DA8: 75 g flaxseed + 800 mg *D. angustifolia* extract/kg, TS8: 75 g flaxseed + 800 mg *T. schimperi*/kg, CD8: 75 g flaxseed + 800 mg *C. domestica*/kg diet, [2]Sum of saturated fatty acids [3]Sum of monounsaturated fatty acids; [4]Sum of polyunsaturated fatty acids.
