# Supplementary material for: Yolk Fatty Acid Content, Lipid Health Indices, and Oxidative Stability in Eggs of Slow-Growing Sasso Chickens Fed on Flaxseed Supplemented with Plant Polyphenol Extracts
Source: Foods. 2023 Apr 27;12(9):1819. doi: 10.3390/foods12091819 (PMC10178081; doi:10.3390/foods12091819)
Supplement: Supplementary file 1 [file foods-12-01819-s001.zip › Table S2.pdf]

Table S2. The effects of feeding plant polyphenol extracts and flaxseed on fat content and fatty acid concentrations in cooked eggs of slow growing Sasso chickens

Fatty acids (mg /g)	[1]Treatments										Random effect	*p*-value
	FS		VE8		DA8		TS8		CD8			
	Mean	95% [LCL, UCL]	Mean	95% [LCL, UCL]	Mean	95% [LCL, UCL]	Mean	95% [LCL, UCL]	Mean	95% [LCL, UCL]		
C14:0	0.79	[0.71, 0.85]	0.81	[0.74, 0.87]	0.79	[0.72, 0.85]	0.83	[0.76, 0.89]	0.81	[0.74 0.87]	non 0	0.8464
C15:0	0.15	[0.13, 0.17]	0.16	[0.13, 0.18]	0.16	[0.13, 0.18]	0.19	[0.16, 0.20]	0.16	[0.13, 0.18]	non 0	0.2132
C16:0	63.9	[61.1, 66.7]	65.6	[62.9, 68.4]	63.7	[61.0, 66.5]	66.4	[63.7, 69.2]	65.6	[62.8, 68.3]	non 0	0.5566
C17:0	0.46	[0.40, 0.52]	0.53	[0.47, 0.59]	0.50	[0.44, 0.56]	0.58	[0.52, 0.64]	0.52	[0.45, 0.57]	non 0	0.1012
C18:0	21.7	[20.6, 22.8]	22.6	[21.5, 23.7]	22.1	[21.0, 23.2]	22.3	[21.2, 23.4]	23.6	[22.5, 24.7]	non 0	0.16807
C20:0	0.02	[0.002, 0.04]	0.04	[0.02, 0.06]	0.04	[0.01, 0.06]	0.06	[0.03, 0.08]	0.05	[0.02, 0.06]	non 0	0.2555
C14:1cis-9	0.16	[0.13, 0.18]	0.16	[0.12, 0.18]	0.16	[0.12, 0.18]	0.17	[0.13, 0.19]	0.16	[0.12, 0.18]	non 0	0.981
C16:1cis-9	7.07	[6.27, 7.87]	6.99	[6 19, 7.80]	6.91	[6.11, 7.71]	7.43	[6.63, 8.24]	7.00	[6.19, 7.80]	non 0	0.8927
C18:1cis-9	104	[99.5, 107]	107	[103.2, 111]	105	[101.0, 109]	104.0	[100.6, 108]	107	[103.6, 111]	non 0	0.54154
C18:1cis-11	4.87	[4.52, 5.22]	4.92	[4.57, 5.27]	4.84	[4.49, 5.19]	5.00	[4.65, 5.34]	4.82	[4.47, 5.16]	non 0	0.9473
C18:1trans-11	0.19	[0.16, 0.22]	0.18	[0.15, 0.21]	0.20	[0.17, 0.23]	0.21	[0.18, 0.23]	0.22	[0.19, 0.24]	0	0.4258
C18:2*n*-6	34.6	[31.2, 38.0]	35.5	[32.1, 38.9]	36.4	[33.0, 39.8]	38.9	[35.5, 42.3]	36.0	[32.6, 39.4]	non 0	0.4476
C18:3n-6	0.17^{a}	[0.14, 0.19]	0.17^{a}	[0.14, 0.19]	0.17^{ab}	[0.15, 0.19)	0.24^{b}	[0.21, 0.26]	0.18^{ab}	[0.15, 0.20]	non 0	0.00111
C18:3*n*-3	6.42^{a}	[5.44,7.39]	7.22^{ab}	[6.25, 8.19]	8.16^{b}	[7.19, 9.12]	8.48^{b}	[7.51, 9.45]	7.99^{b}	[7.02, 8.96]	non 0	0.03423
C18:2 cis-9,trans 11	0.16	[0.12, 0.19]	0.14	[0.10, 0.17]	0.19	[0.15, 0.23]	0.20	[0.15, 0.23]	0.15	[0.10, 0.18]	non 0	0.1365
C18:4n-3	0.04^{a}	[0.02, 0.05]	0.04^{ab}	[0.02, 0.06]	0.06^{bc}	[0.04, 0.07]	0.08^{c}	[0.06, 0.09]	0.06^{bc}	[0.04, 0.07]	non 0	0.00924
C20:2n-6	0.30	[0.25, 0.34]	0.34	[0.29, 0.37]	0.31	[0.27, 0.35]	0.31	[0.26, 0.35]	0.30	[0.25, 0.34]	non 0	0.7123
C20:3n-9	0.05	[0.03, 0.07]	0.04	[0.02, 0.06]	0.04	[0.03, 0.06]	0.05	[0.02, 0.06]	0.04	[0.02, 0.06]	non 0	0.9351
C20:3n-6	0.34^{a}	[0.31, 0.36]	0.34^{a}	[0.31, 0.36]	0.34^{ab}	[0.31, 0.37]	0.39^{b}	[0.36, 0.42]	0.35^{ab}	[0.32, 0.37]	non 0	0.0239
C20:3n-3	0.12^{a}	[0.09, 0.14]	0.16^{b}	[0.13, 0.18]	0.16^{b}	[0.14, 0.18]	0.16^{b}	[0.14, 0.18]	0.15^{b}	[0.13, 0.17]	non 0	0.04234
C20:4*n*-6	4.01	[3.73, 4.29]	3.97	[3.69, 4.25]	3.65	[3.37, 3.93]	4.15	[3.87, 4.43]	3.82	[3.54, 4.10]	non 0	0.1302
C20:5*n*-3	0.17^{a}	[0.13, 0.19]	0.17^{a}	[0.14, 0.20]	0.19^{ab}	[0.16, 0.22]	0.23^{b}	[0.20, 0.26]	0.18^{ab}	[0.15, 0.21]	non 0	0.0165
C22:4n-6	0.43^{ac}	[0.36, 0.50]	0.53^{ab}	[0.46, 0.60]	0.40^{c}	[0.33, 0.47]	0.50^{c}	[0.43, 0.57]	0.41^{c}	[0.33, 0.47]	non 0	0.0397
C22:5*n*-6	0.38	[0.27, 0.47]	0.35	[0.25, 0.45]	0.26	[0.16, 0.36]	0.31	[0.21, 0.41]	0.25	[0.15, 0.35]	non 0	0.3276
C22:5*n*-3	0.83^{a}	[0.63, 1.02]	1.26^{b}	[1.07, 1.45]	1.05^{ab}	[0.86, 1.24]	1.44^{c}	[1.25, 1.63]	1.05^{ab}	[0.86, 1.24]	non 0	0.00095
C22:6*n*-3	6.06^{a}	[5.70, 6.42]	6.74^{b}	[6.38, 7.11]	6.56^{ab}	[6.20, 6.93]	7.45^{c}	[7.09,7.81]	6.67^{bc}	[6.31, 7.04]	non 0	0.00023
[2]∑SFA	87.1	[83.9, 90.4]	89.9	[86.6, 93.1]	87.4	[84.2, 90.6]	90.5	[87.3, 93.7]	90.8	[87.6, 94.0]	non 0	0.32185
[3]∑MUFA	116	[112, 121]	120	[115, 124]	117	[113, 122]	118	[113, 122]	120	[115, 124]	non 0	0.71882
[4]∑ PUFA	54.1	[49.8, 58.4]	57.0	[52.7, 61.2]	58.0	[53.7, 62.3]	62.9	[58.6, 67.2]	57.7	[53.2, 62.0]	non 0	0.08507
∑ *n*-3 PUFA	13.6^{a}	[12.40, 14.90]	15.61^{b}	[14.40, 16.80]	16.20^{bc}	[15.00, 7.40]	17.90^{c}	[16.60, 19.10]	16.10^{bc}	[14.90, 17.30]	non 0	0.00099
∑ *n*-6 PUFA	40.2	[36.70, 43.70]	41.2	[37.70, 44.70]	41.60	[38.10, 45.10]	44.8	[41.30, 48.30]	41.30	[37.80, 44.80]	non 0	0.3988
Fat (%)	25.7	[24.9, 26.6]	26.6	[25.8, 27.5]	26.3	[25.4, 27.1]	27.1	[26.3, 27.9]	26.8	[26.0, 27.7]	non 0	0.18607

Estimated marginal means (emmeans) with 95% upper confidence (UCL) and lower confidence limits (LCL); emmeans within a row with ^{abc}different superscript letters are significantly different at $p < 0.05$. [1]FS: 75 g flaxseed + no antioxidant sources, VE8: 75 g flaxseed + 800 mg α-tocopherol/kg, DA8: 75 g flaxseed + 800 mg *D. angustifolia* extract/kg, TS8: 75 g flaxseed + 800 mg *T. schimperi*/kg, CD8: 75 g flaxseed + 800 mg *C. domestica*/kg diet, [2]Sum of saturated fatty acids [3]Sum of monounsaturated fatty acids; [4]Sum of polyunsaturated fatty acids.
